# Coordinated Care: the new model in primary health care in Poland—Implementation and early trends

**DOI:** 10.3389/fpubh.2026.1737980

**Published:** 2026-04-15

**Authors:** Aleksander Michał Biesiada, Daria Zawodnik, Karolina Kłoda, Mateusz Babicki, Agnieszka Mastalerz-Migas

**Affiliations:** 1 Polish Society of Family Medicine, Wroclaw, Poland; 2 Department of Family Medicine, Uniwersytet Medyczny im Piastow Slaskich we Wroclawiu, Wroclaw, Poland

**Keywords:** Coordinated Care, coordination, general practice, healthcare reform, primary healthcare

## Abstract

**Introduction:**

This narrative review explores the development and implementation of Coordinated Care (CC) within Primary Health Care (PHC) systems in Europe, with a focus on Poland. CC aims to provide continuous, integrated, and patient-centered care, particularly for individuals with chronic conditions.

**Methods:**

By analyzing PHC models across 13 European countries, the review highlights variations in the scope of services, roles of primary care teams, and the integration of diagnostic and specialist support.

**Results:**

In Poland, a CC model was introduced nationally in 2022 following a successful pilot. It emphasizes enhanced roles for general practitioners, nurses, and care coordinators, and includes comprehensive consultations, individual health care plans (IHCPs), and expanded diagnostic access. Data from public sources from 2023 to 2025 show growing provider participation and improved diagnosis rates, particularly for chronic kidney disease.

**Discussion:**

The Polish model demonstrates that systemic reforms based on PC team collaboration and fee-for-service financing can be a way of strengthen PHC systems by better resource utilization. While early results are promising, further evaluation is needed to assess long-term outcomes and guide adaptations in other healthcare settings.

## 1 Introduction

The aim of this paper is to examine Coordinated Care (CC) within Primary healthcare (PHC) systems across Europe, with a specific focus on Poland, including exploration of the advantages of Poland's new model of care in comparison to those of other European countries, as well as deriving implications for other European countries. The paper will describe the implementation of CC, using Poland as a case study, highlight the challenges faced during this implementation, and explore the expected outcomes. Additionally, this paper aims to evaluate Poland's novel approach to CC and compare it with the models implemented in other European countries, offering insights that could benefit other healthcare systems aiming to introduce similar reforms. Comparison between the CC model of care with the actual implementation within a single healthcare system provides valuable insights into the topic.

CC in PHC is the integration and collaboration to deliver patient-centered, continuous, and efficient care. It involves seamless communication between various professionals to address all aspects of a patient's health needs. This approach ensures comprehensive, personalized care with no gaps, improving overall health outcomes. According to the World Health Organization, one of the core characteristics of PHC is the coordination of patient care (1). In many countries, efforts are underway to enhance PHC to provide comprehensive and cohesive care to patients. In former Eastern Bloc countries, such as Hungary, Slovakia, and the Czech Republic, PHC reforms have been prominent on political agendas. Hungary's CC initiative, introduced in 1998, stands as a pioneering example of CC in Europe (2–5). However, in countries like Slovakia and the Czech Republic, there has been a decline in the role of PC teams, which risks diminishing access to quality care, especially for vulnerable populations with limited financial resources.

Modern healthcare systems face increasing challenges due to patients' complex health needs, comorbidity, and the aging population, especially in the northern hemisphere. Balancing patient autonomy in accessing specialized and hospital care with the capacity of healthcare systems is an ongoing challenge (6). One approach to addressing this challenge is through the coordination of healthcare services, underpinned by robust PHC systems.

The goal of CC is to improve the quality, efficiency, and continuity of care, particularly for patients with chronic conditions. CC facilitates easier access to healthcare services, better continuity of care, and more effective treatment across various stages of a patient's health journey. In Poland, a novel CC model was introduced at the PHC level in 2022, offering valuable lessons for other European countries considering similar reforms.

## 2 Methods

**Figure 1:**
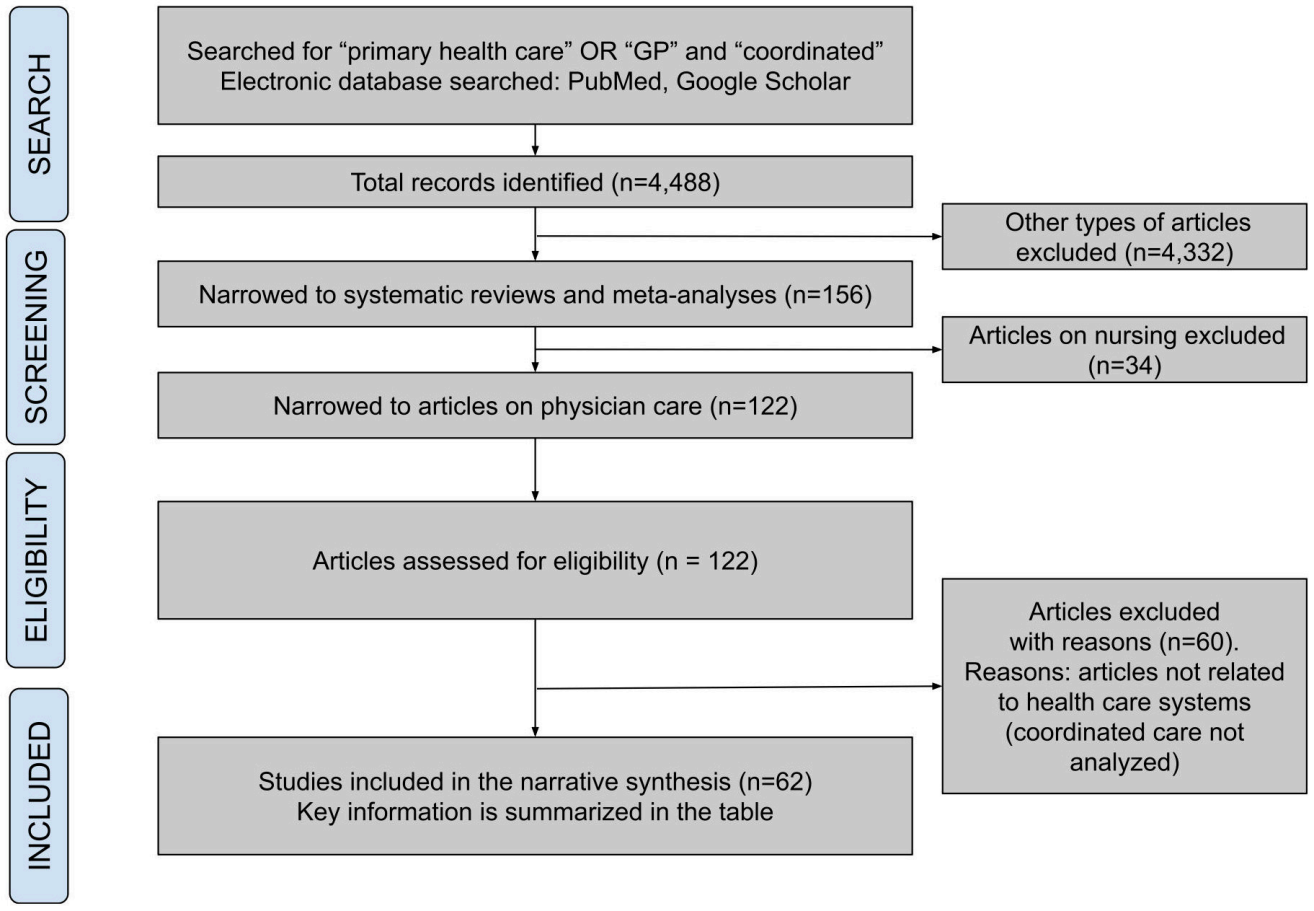
Narrative review selection process of the literature.

A literature review on CC in PHC was conducted using the following key terms: “primary health care” OR “GP” and “coordinated” by searching PubMed and Google Scholar scientific article databases. Inclusion criteria for articles were: publications issued after 1990 (major political changes in the number of European countries) related to CC and concerning European countries. The initial yield resulted in approximately 4,488 articles, which were then screened for relevance based on predefined inclusion and exclusion criteria. Both empirical studies, systematic reviews, review articles, and reports describing the concept, efficiency evaluation, or results of PHC systems in individual countries were analyzed. Articles had to be available in English or Polish. Exclusion criteria were: publications not related to PHC, popular science articles, conference materials. Additionally, articles not available in full text and written in languages other than Polish or English without access to full translation were excluded from the analysis. A critical assessment of the articles' content was conducted, taking into account their titles, abstracts, and full text. Articles that did not answer the research questions or did not meet the inclusion criteria were excluded from the analysis. No formal quality appraisal of the included studies was conducted in this review. This decision was based on the scope of the review and the focus on a broad range of studies. Authors focused on the relevance and comprehensiveness of the studies rather than their individual methodological quality. The process is presented on Figure 1. In addition, data from public sources in Poland were used, including data from the National Health Fund and the Ministry of Health.

## 3 Results

### 3.1 Narrative review over CC in Europe

CC foundation is a paradigm shift from episodic assistance to long-term and preventive approaches (7). CC services include diagnostics, consultations, treatment and continuous follow up of patients and it is the crucial part of PHC. While introducing the topic of CC in should be noted that the strength of evidence on CC varies significantly across countries, with peer-reviewed studies generally providing more rigorous and reliable data compared to policy reports. For instance, countries like Germany and the Netherlands have robust, empirical studies with large sample sizes and longitudinal data, demonstrating the effectiveness of CC in improving patient outcomes and system efficiency. In contrast, some countries, such as Slovakia and the Czech Republic, rely more on policy reports and pilot project evaluations, which, while valuable, often lack long-term follow-up and detailed statistical analysis. As a result, the evidence from these countries is less conclusive, making it difficult to assess the full impact and sustainability of CC models. Based on the literature meeting the criteria for inclusion in the narrative review, a comparison of PHC systems in 14 countries was made. In 11 of them, the system meets the criteria for CC, in one (Belarus) the data are uncertain (the country has started changes in the PHC system). In the Czech Republic, the system does not meet the criteria for the definition of CC, while in Lithuania the model is partially met (8, 9).

In all countries where CC has been implemented, its introduction was a gradual process (2) as demonstrated in the Hungarian example, with systemic changes introduced in 1998 and further updated in 2017 (2, 10–12). Also Germany, where patients are encouraged to join Disease Management Programs (organized care for patients with chronic diseases) by insurance funds through benefits is a good example (13–15). Care coordination was carried out either at the level of PC facilities or at local centers, as seen in Austria (16, 17) or Greece (18–21). In some countries, such as Belgium (6, 8, 22, 23), the healthcare system emphasizes patient independence and freedom, allowing direct access to specialist care. However, even in these systems, patients with chronic conditions have established care pathways that involve collaboration among the patient, the PC physician, specialists, and other healthcare professionals. A similar approach is found in France, where care pathways not only support patients with chronic illnesses but also include individuals over 75 who are at risk of dependency (8, 24). Also Swedish primary care centers employ diverse healthcare professionals, and since 2019, coordinated, patient-centered care pathways and joint regional-municipal plans have aimed to enhance care integration and prevent delays (25, 25–28).

Finland serves as an example of a country that recognizes the essential role of PC in providing CC for patients with chronic conditions (8, 29). However, if costly procedures are needed, a specialist consultation is required.

The essence of CC lies in transitioning from a model where GPs work solitarily to one where they are integrated into a PC team, collaborating closely with other specialists. Italy is following this approach (8, 30, 31). The Chronic Care Model also allows for better management of chronic diseases.

An advanced model of CC that takes into account responsibility for continuity of care, cooperation with specialists, telephone consultations and follow-up exists in the Netherlands (8, 32, 33). Many countries include advanced care planning in their CC models. For instance, Norway's 2012 reform introduced care planning specifically for patients with chronic conditions (1, 34, 35).

In systems without CC, key limitations include restricted access to specialist consultations, limited diagnostic options, and the inability to prescribe therapies aligned with the latest scientific guidelines without specialist consultation, as seen in the Czech Republic (2, 8, 36). The family doctor either solely functions as a gatekeeper or does not even hold this role at all (partially in Belarus) (37).

Key facilitators of CC implementation include the integration of primary care teams, with general practitioners, nurses, and care coordinators playing central roles in care coordination. Digital tools and telemedicine have expanded access, especially in underserved areas, as seen in countries like Belgium and Finland. Financial incentives, such as fee-for-service or capitation-based models, have supported service expansion and sustainability, particularly in Poland's CC model. These elements have collectively improved care continuity and patient satisfaction.

Workforce shortages, particularly in family physicians and specialists, are a major barrier to CC implementation, leading to increased provider burden and burnout, as reported in countries like Slovakia and the Czech Republic. Financial sustainability remains a concern, with fee-for-service models in some countries failing to secure long-term funding for CC. Additionally, access to services is inequitable, with vulnerable populations in rural or low-income areas facing challenges in utilizing CC, as seen in Italy and Spain. These barriers highlight the need for more comprehensive solutions to ensure effective and equitable CC delivery.

The most important details of the CC model for chosen European countries, identified due to literature search are presented in Table 1. This table collectively presents the role and scope of the PHC, including CC, describes the competences of a family physician and the role of a nurse.

**Table 1:** European countries PHC model according to coordination of care model.

Country	Coordinated care model features	Details	Competencies of family doctor	Role of a nurse
Austria	Yes	The Austrian primary healthcare system is evolving toward the creation of new, multidisciplinary centers to better meet the needs of society, especially for people with chronic diseases. In 2005, a reform recognized the need for nationwide standards for chronic care and a year later health funds were established in each of the Austrian states. Since 2008, the Competence Center for Integrated Care (CCIV) has been involved in the development of a structured approach to disease management. These projects include the DMP for type 2 diabetes, the development of interdisciplinary guidelines for the treatment of chronic diseases, and the development of integrated care for people with dementia	Limited role of family doctors, patients often go straight to other specialists	Sometimes the standard model of single-person primary care medical practices also includes nurses
Belgium	Yes	Goal-oriented model. This model of primary care allows patients to have greater freedom to choose their care providers, get a second opinion or even consult with multiple specialists at the same time. Patients also have direct access to specialist care, which contributes to the flexibility and speed of healthcare delivery. In 2009, care pathways for patients with chronic diseases such as diabetes and chronic renal failure were introduced, aimed at organizing collaboration between patients, primary care physicians, specialists and other healthcare professionals. This initiative has improved care processes and patient satisfaction, but its use has not been optimal on a national scale due to a lack of awareness of its existence by both medics and patients	Broad	Wide, including care for people with chronic diseases or disabilities, e.g. wound care and care for patients with diabetes
Belarus	Lack of data	Primary health care does not fulfill its gatekeeping role, especially in cities, where it is replaced by specialists gathered in polyclinics. In November 2016, the “Health System Modernization” project was launched, which aims to accelerate the diagnosis process and reduce the need for hospitalization of patients		
Czech Republic	No	Patients have direct access to family doctors, nurses and physiotherapists working in primary care, and do not need referrals to visit gynecologists, pediatricians, ophthalmologists, otolaryngologists or surgeons. Specialists always communicate after consultation with the referring family doctor	Broad	Limited
Finland	Yes	The primary care system includes health centers and occupational health units. Most chronic diseases are treated by general practitioners, but there are situations where specialist care is necessary, especially in the case of new and expensive therapies	Broad	Wide, model in which a nurse conducts an initial assessment of the patient and later consults a doctor depending on the needs is increasingly common
France	Yes	Initiatives have been introduced to improve coordination and continuity of care, such as the gatekeeping structure or the development of care pathways for patients with chronic diseases and those over 75 years of age at risk of dependency. The gatekeeping system introduced in 2005 is mainly based on the possible imposition of financial penalties, and a referral is not required to see specialists such as pediatricians (for patients under 16 years of age), gynecologists, ophthalmologists and psychiatrists (for patients under 25 years of age)		There are no advanced roles for nurses in primary care
Germany	Yes	Since 2004, insurance funds are required to offer policyholders the opportunity to enroll in a GP-oriented model, and some even offer bonuses for following the “helowingedrs rules. In 2003, Disease Management Programs (DMPs) were introduced, aimed at providing organized care for patients with chronic diseases. After enrolling in the program, patients receive detailed information brochures that describe the treatment of a given chronic disease and present key aspects of the program, and the doctor and patient establish therapeutic goals. Participation in a DMP is voluntary, but insurance funds can additionally encourage participation by offering various benefits. The procedure of referring the patient to a specialist can be initiated in the case of unsatisfactory treatment results or the need to change the therapy to a more effective and long-term one	Broad	Minor role. There is a trend toward practices with multidisciplinary cooperation, but single-handed practices predominate. There are no medical centers in which nurses coordinate care.
Greece	Yes	As part of the primary care reform, a law was passed to establish family doctors as a “gateway” before patients go to specialists. The reform included the introduction of local health units (TOMY) and an integrated family doctor scheme to provide holistic and patient-centered care. Greece struggles with high rates of chronic disease, and integrated care is still in its early stages of development. Reforms to the primary care system are met with gaps in public health and health promotion	Broad	Nurses are part of multidisciplinary health teams
Hungary	Yes	The scope of primary care physicians' activities is wide and includes the management of chronic diseases, palliative care, involvement in antenatal care and vaccinations. Unfortunately, preventive services are not properly implemented, visits to doctors are most often due to chronic diseases or acute complaints. In 1998, an innovative Care Coordination System (CCS) was introduced to optimize patient paths and save costs. Care providers had the ability to manage a virtual budget, and disease management programs were integrated within the CCS. The reform of primary health care contin ued since 2017 includes the integration of family doctor practices into larger groups, additionally involving other professionals (dietitians, physiotherapists)	Broad	Limited. Highly qualified nurses provide patient education and consultation services
Italy	Yes	In recent years, the primary care system in Italy has undergone a significant metamorphosis, with the traditional model of solo practices gradually giving way to new organizational forms such as networks or groups. Primary care physicians cannot directly prescribe certain categories of medications, and a form must be completed by a specialist. CCM (Chronic Care Model) aims to improve the health status of patients through proactive, planned and population-focused care. The Italian “National Chronicity Plan” defines CCM as the reference organizational model for the management of chronic diseases. The ex tent to which this system is implemented varies across different regions	Limited	Since 2005, in some regions (e.g. Emilia Romagna, Lombardy, Piemonte, Veneto and Toscana) there have been programmes to develop organizational models integrating different professionals, including nurses, to provide comprehensive care outside hospitals. Nurses are also involved in palliative care
Lithuania	Yes	Efforts are being taken to strengthen the role of primary health care, particularly in the areas of disease prevention and the management of chronic diseases. Since 2002, family doctors have been fully gatekeepers, with their responsibilities including diagnosis and follow-up care. Family doctors also provide preventive care, screening tests, and individual health advice. In the case of certain chronic diseases (e.g. diabetes, circulatory system diseases), the primary care physician is obliged to refer the patient to a specialist once a year		Since 2015, nurses have been able to prescribe medical aids (under the supervision of a physician) and have been given a greater role in the management of patients with chronic non-communicable diseases (lifestyle advice, self-care and health monitoring during check-ups)
Netherlands	Yes	GPs play a key role as gatekeepers in the healthcare system, referring patients to specialists and coordinating their care. Care for patients with chronic diseases is provided by care groups that take clinical and financial responsibility for these patients, enabling integrated care and effective treatment management. In the Netherlands, there is also cooperation between GPs and specialists, including clinical lessons and telephone consultations. GPs are often responsible for the follow-up care of type II diabetes, mild depression, palliative cancers, or congestive heart failure. They are involved in preventive care and health promotion advice	Broad	Primary health care includes home nurses, specialist nurses, and primary care nurses
Norway	Yes	The 2015 White Paper proposed a reorganization of primary care by establishing interdisciplinary primary care teams, which include teams providing general community health care and chronic disease care teams. Since the 2012 healthcare coordination reform, there has been a greater emphasis on the responsibility of municipalities for 24-hour care and after hospital discharge. These include, among others, the obligation to establish individual treatment plans for patients with chronic diseases. In 2019, Healthcare Communities were introduced, which improved cooperation between municipalities and specialist care	Broad	Nurses are responsible for the care of children from birth to age of 12 as part of the national vaccination programme and regular check-ups. General nursing services are available to all residents of a municipality. These include social care homes and home care for those in need. Municipality nursing service contact patient's GP in case of the need for consultation or referral to specialist as a part of coordination patients' home care
Portugal	Yes	In 2005, a reform of primary health care was introduced by creating public, self-governing family care units (USF - Unidades de Saúde Familiar). They have autonomy in organizing health care and operating procedures. The reform introduced better health information systems, goal-oriented management and reward for performance. Avoided hospitalization rates in Portugal are among the best in the OECD for chronic diseases, partly due to the introduction of commissioning processes and the promotion of safe prescribing		Nurses are part of USF. Nurse-led substitution of care is very common, especially in health education and prevention. They are involved in antenatal care at primary care level, regular check-ups of children and implementation of the National Vaccination Plan
Slovakia	Yes	Since 2013, patients have not been able to use specialist services without a referral from their GP, unless there are emergencies or a need for a psychiatric, gynecological, dermatological, ophthalmological or dental consultation. The reforms to the Slovak healthcare system, presented in 2013, focus on early detection and effective treatment of chronic diseases in primary healthcare. These plans propose services including screening, physical therapy, dietary counseling, dental and gynecological care. In 2014, a plan for the reform of primary healthcare was introduced, which assumed the creation of larger integrated care teams (ICCs), bringing together different health specialists in one place. However, progress in implementing these plans has been slow due to the lack of concrete implementation	Broad	Limited It is very rare for nurses to run specialist clinics (e.g. diabetic) or provide health education
Sweden	Yes	Swedish primary care centers (PHCCs) employ multi-professional staff PHin addition to general practitioners and nurses, these include physiotherapists, social workers and occupational therapists. In 2019, coherent and patient-centered care pathways were introduced, which are intended to improve care coordination and prevent delays in diagnosis and treatment. Regions and municipalities are required to cooperate to facilitate this process, including by creating a so-called coordinated individual plan	Broad	Nurses and nurse assistants are part of the multi-professional team of Swedish primary care centers (PHCCs). Nurse-led diabetes clinics and health education are very common
The UK	Yes	Coordinated Care in PHC is provided through Integrated Care Systems (ICS) and Primary Care Networks (PCNs). These initiatives aim to integrate services across healthcare sectors, including GP practices, hospitals, social care, and community services. The focus is on improving patient outcomes through collaboration, personalized care, and multidisciplinary teams. ICS and PCNs work to offer more patient-centered care and better access to services, especially for those with complex or chronic conditions	Broad	Services in GP practices, managament of chronic diseases, promotion of health and prevention, health education. Working in multidisciplinary teams to support complex patient needs. Advanced Nurse Practice to ensure quality of care

### 3.2 Narrative review over polish PHC

#### 3.2.1 PHC from 1989 to 2022 (Before CC)

Polish healthcare has changed significantly over the last three decades in terms of quality, financing, and management. Before 1989, when Poland was part of the communist bloc, the healthcare system was modeled on the Soviet model (the so-called Semashko model). After the fall of communism, healthcare reform first introduced Health Funds, which were later replaced by the Voivodeship Branches of the National Health Fund (NHF). NHF is a public institution responsible for overseeing and financing healthcare services. It is funded by contributions from both employees and employers, as well as state budget revenues. Health insurance is universal.

Similar changes also affected PHC. In the early 90's due to the World Bank project, a group of doctors and scientific workers from Poland traveled to the Netherlands to get acquainted with the idea of family medicine. After their return, public district healthcare institutions were transformed into units dependent on local governments or private entities. The care model was based on family medicine specialists with first family doctors practices functioning from 1995.

#### 3.2.2 PC services

Currently PC is the first point of contact for patients and plays a crucial role in the Polish national healthcare system. There are 43 000 medical doctors working in PCs (38). Only 12 000 of them are family medicine specialists (39), although systematic actions are taken to increase this number. The rest are internal medicine specialists, pediatricians, doctors of other specialties and doctors without specialization (also family medicine residents).

The functioning and financing of PC is based on a capitation rate calculated by the number of patient declarations submitted to the doctor and nurse, and in the case of women, to the PC midwife. Based on the number of patients declared to a given facility, the National Health Found (NHF) provides financing. A capitation rate multiplier applies to patients from extreme age populations (children and older adults), and PC also receives additional remuneration depending on the number of chronic diseases occurring in a specific patient. In addition, selected, individual laboratory tests are financed from a special entrusted budget.

Within PC, patients can visit the unit, have home visits, and receive teleconsultations (with restrictions). Guaranteed services by a PC physician include medical consultations, children's preventive check-ups, ensuring appropriate diagnostics, providing cardiovascular disease prevention services, and vaccinations. Guaranteed PC services also include nursing and midwifery care and sanitary transport (40). PC physicians can order diagnostic tests financed from a capitation fee and, separately from a special entrusted budget, in which payment is made on a fee-for-service basis. In most cases, it is the PC physician who decides on the initiation and/or continuation of diagnostics and treatment within ambulatory specialist care (ASC) by issuing an appropriate referral (in strictly defined cases, it is not required) and on hospital treatment (outside life-threatening conditions requiring immediate assistance).

#### 3.2.3 PC physician responsibilities

To fully understand PC roles it is important to notice the WHO definition of PC. At the same time the scope of activities of Polish PC physicians is broad. That is why we place the CC in Poland in the context of PC physicians' roles. Those tasks include a wide range of activities from prevention, diagnosis, to treatment and referrals to ASC, including rehabilitation (also sanatorium) and issuing certificates in selected cases. In Poland, the scope of tasks for a PC physician is defined in the regulation of the Minister of Health (41).

[1] In the area of health promotion the PC physician undertakes educational activities to help patients understand the importance of a healthy lifestyle and the benefits and necessity of prevention. They regularly conduct health assessments during preventive check-ups in children and follow-up visits, allowing early detection of potential diseases. [2] In the area of prevention, the doctor identifies risk factors and health threats, taking actions to minimize them. They provide information, conduct qualification exams, and coordinate vaccinations. They also participate in preventive programs and screenings that allow early detection of diseases. [3] Diagnosing diseases is another important area of the PC physician's work. They plan and coordinate diagnostic procedures depending on the patient's health status. They conduct medical interviews, perform physical examinations, and, if necessary, order additional tests. In the case of suspected malignancy, the PC physician issues an oncology diagnostic and treatment card, directing the patient for further specialist examinations. [4] In treating diseases, the PC physician plans therapeutic procedures, monitors pharmacological treatment, and orders necessary procedures. If needed, they refer the patient to other providers of ambulatory or inpatient health services. The doctor can also refer the patient to long-term nursing home care. [5] Another aspect of the doctor's work is certifying temporary incapacity to work or study and issuing medical certificates. [6] In the area of rehabilitation, the PC physician issues referrals for physiotherapeutic procedures and prescribes patients the necessary medical supplies (41).

#### 3.2.4 Pilot program

The beginnings of CC in Poland date back to 2017, when the Minister of Health's regulation initiated the pilot program “POZ-Plus” (PC-Plus) (42). The project covered about 40 PC clinics and 350,000 people in Poland.

The PC-Plus project was broadly evaluated (42). The results highlighted positive aspects of its implementation, such as an increase from 79 to 100% in the number of service providers monitoring the health status of patients, an increase in the use of tele informatics resources, or an increase in the maturity of PC through the implementation of periodic meetings where the situation of patients is discussed (from 58 to 92%). Analyzing for example the services provided to patients at other levels of care, it can be stated that patients with type 2 diabetes were provided with fewer services outside the program after the implementation of the PC-Plus (43). Potential difficulties were also revealed, for example the fact that despite receiving care under PC-Plus, patients continued to receive specialist care outside the programme. There was also no statistical significance in the differences in the dimensions of care indicated by the patient, except for one. Within the PC-Plus, the specialist's knowledge of the patient was higher.

In response to the need to improve the effectiveness of care for an increasing number of patients with chronic diseases and multimorbidity, a CC system was introduced. Following the pilot, on September 15, 2022, the Minister of Health issued a regulation that assumed the implementation of CC in PHC from October 1, 2022.

### 3.3 CC and its implementation in Poland

#### 3.3.1 CC in PHC in Poland

The implementation of CC is a process currently underway, and PC work in this model voluntarily. Forty-nine percentage of of PC providers implementing this care model at 12th December 2025 (44).

CC services include diagnostics and treatment, both in adults and children of: hypertension, heart failure, chronic coronary artery disease, atrial fibrillation, pre-diabetic conditions and diabetes, asthma, and chronic obstructive pulmonary disease (COPD, emphysema and chronic bronchitis, hypo- and hyperthyroidism, and the diagnosis of single and multiple thyroid nodules, as well as chronic kidney disease (CKD) (added to the CC in 2023) (45–48).

**Figure 2:**
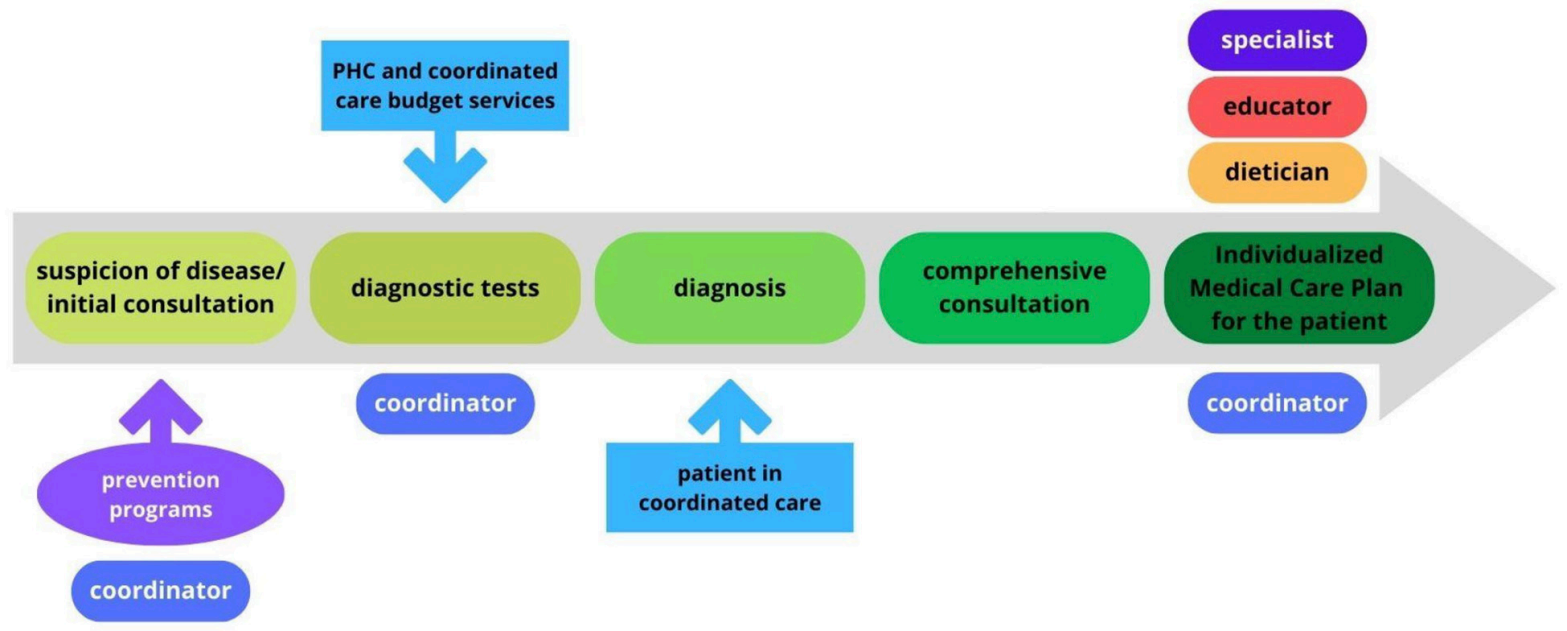
Flowchart of CC model in Poland.

The model is based on a PC team. The PC physician is the decision-maker regarding necessary interventions; the nurse takes on more responsibilities in disease prevention and education, and the coordinator is responsible for administrative tasks, planning diagnostics and consultations, inviting patients for examinations, and coordinating communication between patients and other staff and collaborating entities. The patient pathway through the CC model is shown on Figure 2.

CC strengthens the role of the family physician. In the areas covered by the CC, the diagnostic possibilities of PC have been expanded, which gives the doctor a chance to actively search for and manage chronic diseases. Direct consultation with specialists also supports this approach without the necessity of the referral. Stable patients not requiring additional specialist interventions remain under the care of the PC physician.

#### 3.3.2 The CC tools

CC involves moving away from financing providers primarily through capitation rates. The budget entrusted to CC is a “fee-for-service” (FFS) financing model using rates for specific services determined by the NHF President's Order (Table 2) (48). Services are financed up to the limit calculated for a given facility, with the possibility of reimbursement by the NHF for over-performed services. Financed services within the entrusted budget include specified diagnostic tests, comprehensive consultations, educational and dietary consultations, and consultations with selected narrower field specialists.

**Table 2:** Coordinated care pathways with ICD-10 and new diagnostic tools.

Coordinated care pathways	Available services
Cardiology pathway	
– I10 Essential (primary) hypertension – I11 Hypertensive heart disease – I12 Hypertensive renal disease – I13 Hypertensive heart and renal disease – I15 Secondary hypertension – I20.1 Angina pectoris with documented spasm – I20.8 Other forms of angina pectoris – I20.9 Angina pectoris, unspecified – I25 Chronic ischaemic heart disease – I48 Atrial fibrillation and flutter – I50 Heart failure	Diagnostic tools: – Exercise ECG (stress test ECG) – Holter ECG (24, 48 or 72-hour) – ABPM (24-hour) – Doppler ultrasound of the carotid arteries – Doppler ultrasound of lower extremity vessels – Transthoracic echocardiography (TTE) – BNP (NT-proBNP) – Albuminuria – UACR
Diabetology pathway	
– E10 Type 1 diabetes mellitus – E11 Type 2 diabetes mellitus – E13 Other specified diabetes mellitus – E14 Unspecified diabetes mellitus – R73 Elevated blood glucose level – R73.0 Abnormal glucose tolerance test	Diagnostic tools: – Albuminuria – UACR – Doppler ultrasound of lower extremity vessels
Endocrinology pathway	
– E01 Iodine-deficiency-related thyroid disorders and allied conditions – E02 Subclinical iodine-deficiency hypothyroidism – E03 Other hypothyroidism – E04 Other nontoxic goiter – E05 Thyrotoxicosis [hyperthyroidism] – E06 Thyroiditis – E89.0 Postprocedural hypothyroidism	Diagnostic tools: – Targeted fine-needle aspiration biopsy of the thyroid (in adults) – anti-TPO – anti-TSHR – anti-TG
Pulmonology/Allergology pathway	
– J41 Simple and mucopurulent chronic bronchitis – J42 Unspecified chronic bronchitis – J43 Emphysema – J44 Other chronic obstructive pulmonary disease – J45 Asthma	Diagnostic tools: – Spirometry – Spirometry with a bronchodilator reversibility test
Nephrology pathway	
– N18 Chronic kidney disease	Diagnostic tools: – UACR
Available for each pathway	
– Comprehensive consultation with the development of an Individual Health Care Plan (IHCP) – Specialized consultations – Educational consultations – Dietary consultations	

The primary goal of the CC model is to provide patients with comprehensive care, which translates into greater availability of diagnostic tests, educational and dietary consultations, and specialist consultations when needed, both through direct contact between the doctor and patient and through consultations between the PC physician and a narrower field specialist. Specialist consultations can be carried out both in a stationary form with patient-specialist contact and through teleconsultations between the family doctor and the specialist.

Within CC, each patient can benefit from nine consultations (educational and/or dietary) per year. Educational consultations are conducted by a nurse or PC physician, with the number, frequency, and scope of education individually determined after assessing the patient's needs and expectations in this regard. During each consultation, the educator also assesses the patient's health status. Dietary consultations provided by qualified dietitians are similarly individualized.

A key supporter in care for both medical staff and patients is the coordinator. The coordinator monitors the proper course of the patient's diagnostic-therapeutic process, ensures proper medical documentation flow, and facilitates better communication between the patient and medical and administrative staff. In many facilities, the coordinator is also responsible for contacts with subcontractors and external partners collaborating with the facility within CC. Coordinator's duties also include implementation of preventive programs. The coordinator's work is financed within the capitation rate.

#### 3.3.3 Individual healthcare plan (IHCP)

One of the key elements of CC is the possibility of conducting comprehensive consultations with the development of an Individual Health Care Plan (IPOM, eng. IHCP). The PC physician caring for a patient within CC is obliged to conduct one comprehensive consultation annually, during which the patient should undergo an examination, an analysis of test results and applied treatment, and further actions should be determined. The result of the comprehensive consultation is the creation of an IHCP, a document in the form of Electronic Medical Records containing guidelines for the next 12 months. The patient, supported by the coordinator, is responsible for implementing the IHCP. It can be modified, for instance, in the case of the need for additional tests, the diagnosis of a new disease unit, or changing the PC clinic. The IHCP should include information on planned visits and check-ups, pharmacological and non-pharmacological management, educational and dietary consultations, specialist consultations, and additional recommendations, such as vaccinations.

#### 3.3.4 CC implementation in Poland (Q4-2022 to Q4-2025)

Based on an analysis of data from the NHF for 2022, clear trends can be seen regarding the areas of work of PC teams and the ways patients use health services. Three main groups of reasons for visits to PC dominate: [1] chronic conditions such as hypertension, [2] acute upper respiratory infections and acute rhinitis, and (3) repeated prescription issuance (49).

Eleven chronic diseases are most commonly encountered by PC physicians in their practice and account for more than half of all chronic diseases reported at the PC level. These conditions include hypertension, type 2 diabetes, chronic coronary artery disease, permanent atrial fibrillation, asthma, chronic obstructive pulmonary disease (COPD), diffuse and nodular goiter of the thyroid gland, hypothyroidism, osteoarthritis of peripheral joints, and spinal pain syndromes (50). Nine of these diseases qualify the patient for CC.

It is noteworthy that despite 9 million patients with obesity, this condition is not reported from PC and is not included in the payer's lists. It is also not part of CC.

Additionally, the percentage of people using PC increases with age (and increased comorbidity) (49). Since the COVID-19 pandemic, the number of teleconsultations has increased, with local variations (49).

**Figure 3:**
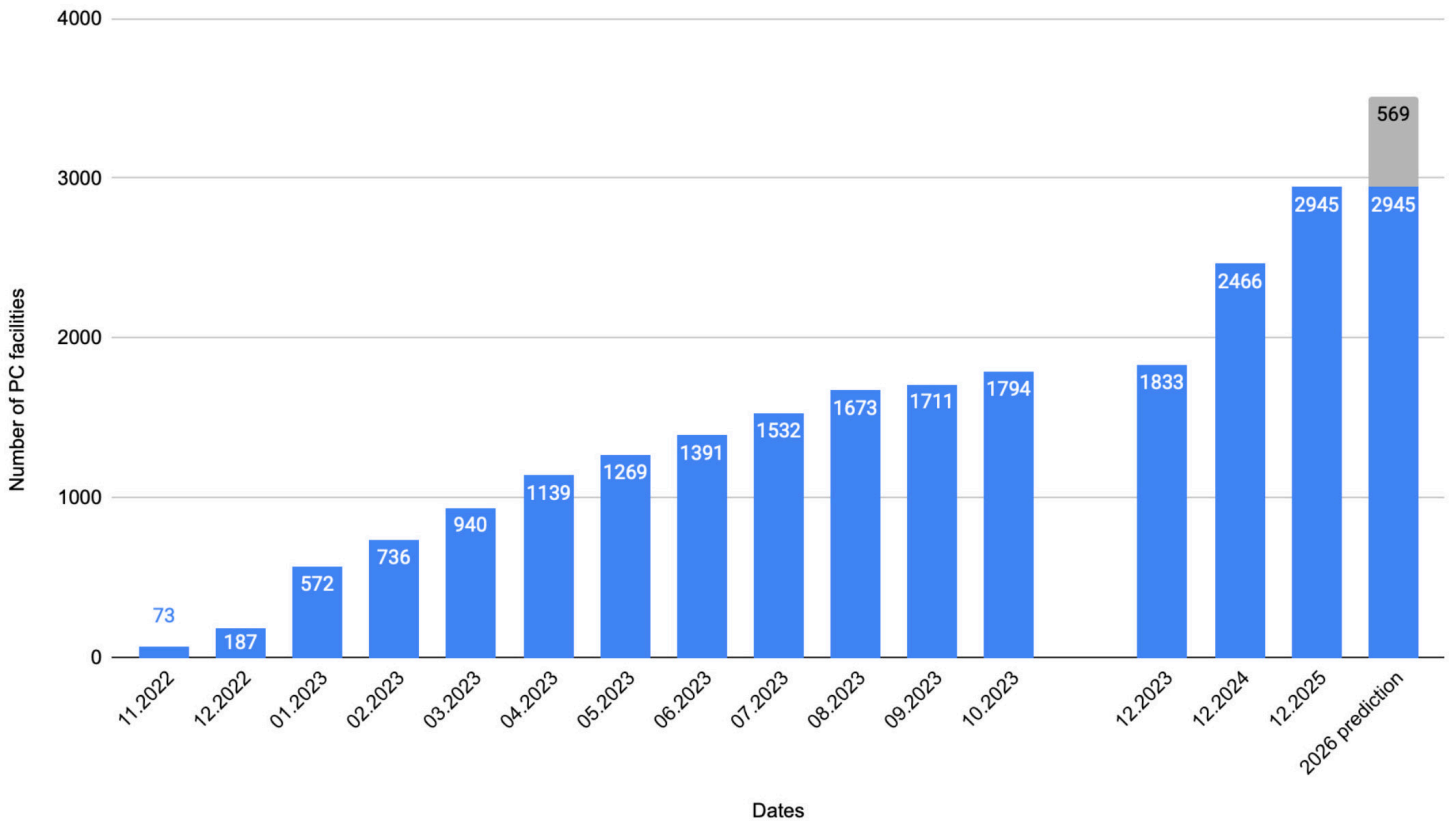
Number of healthcare providers implementing coordinated care between october *2022* and December *2025 with forecast*.

The Figure 3 compares the data on the increase in the number of entities implementing CC. Based on the applications submitted by the PC facilities to the NHF before the end of December 2025, a trend for 2026 was also established.

Based on the data on the implementation of services within the entrusted budget of CC, key areas of PC physicians' work were identified. These include the preparation of IHCP (a total of 593,447 IHCPs for 2023 and 1,164,907 IHCP for 2024) (51). The data analysis shows that PC physicians are largely prepared and independent in providing medical care (diagnostics and treatment). Within CC, they consulted mainly with specialists such as cardiologists and endocrinologists (Supplementary Table S1). The study included results for the full year of 2023 (the first full calendar year after the reform came into effect). The period from October 2022 to December 2022 was excluded as it was when the Ministry of Health, NHF, and PC providers were preparing to contract the service in the new model of CC.

The role of PC physicians in the CC system also includes diagnostics and monitoring of diseases. Key diagnostic tests ordered by PC physicians within CC include ABPM and 24-hours ECG monitoring, cariological ultrasound (ECHO), BNP (NT-pro-BNP), UACR (urine albumin/creatinine ratio), albuminuria, anti-TPO, anti-TG, anti-TSHR, and Doppler ultrasound of the carotid arteries. The number of tests is listed in Table 3.

**Table 3:** List of 10 most frequently settled tests from the coordinated care budget in 2023-2024.

Diagnostic tests	Number of tests in 2023	Percentage of the population with comprehensive consultation in 2023	Number of tests in 2024	Percentage of the population with comprehensive consultation in 2024
ABPM (24-h)	90,244	14.99%	207,579	17.82%
Transthoracic echocardiography (TTE)	69,880	11.61%	179,385	15.40%
Holter ECG (24, 48 or 72-h)	44,656	7.42%	127,400	10.94%
UACR (urine albumin/creatinine ratio)	41,746	6.93%	111,622	9.58%
BNP (NT-proBNP)	30,237	5.02%	81,085	6.96%
anti-TPO (antibodies against thyroid peroxidase)	29,448	4.89%	67,411	5.79%
anti-TG (antibodies against thyroglobulin)	26,169	4.35%	61,124	5.25%
Doppler ultrasound of the carotid arteries	20,861	3.47%	52,918	4.54%
albuminuria	17,617	2.93%	39 399	3.38%
anti-TSHR (antibodies against TSH receptors)	11,642	1.93%	30,672	2.63%

## 4 Discussion

### 4.1 Polish model of CC in Europe

Comparing the work of PC physicians in Poland and other European countries, it can be seen that the main tasks are similar across all countries. PC physicians play a key role as the gatekeepers, that is: first point of contact for patients, providing essential PC, diagnosing diseases, treating them, and referring patients to specialists when necessary. In each country, PC physicians are largely involved in diagnosing and treating chronic diseases. However, their responsibilities and the utilization of their competencies vary. The example of Poland shows that expanding access to diagnostic tools along with transferring responsibility for establishing a care plan for patients with chronic diseases activates PC physicians in the treatment and diagnosis of patients. This must be accompanied by financial resources.

There are differences in the responsibilities of PC team members resulting from differences in healthcare systems and the preferences and needs of patients in individual countries. In the Czech Republic, PC physicians deal not only with basic health care aspects but also conduct screenings for sexually transmitted diseases and provide assistance with selected psychiatric and psychological issues. Danish doctors are involved in reproductive health care for pregnant women (8). In Portugal, PC physicians are engaged in prenatal care, family planning, and perinatal care (52–55). In the UK the fundaments of PHC are integration of care and Advanced Nurse Practice (56). The areas of work can also differ within a single country—for example, in Hungary, minor surgical procedures are often routinely performed by PC physicians in rural offices, while in cities, patients requiring such services are referred to surgeons (12). However, the most common long-term conditions are diagnosed and treated in PC in most countries.

### 4.2 CC implementation

Due to the necessity of contracting facilities by the NHF for CC implementation in the first months after implementation (November-December 2022), service provision was minimal. A real increase in the number of facilities and the implementation of CC services was observed from January 2023, when 572 PHC facilities were contracted. The NHF data indicate that by the end of 2023, 1,833 PHC facilities had signed contracts for the entrusted budget of CC, representing about 30% of all PC facilities in Poland. Forty five percentage of providers implement 4 or 5 domain pathways, from which the last pathway (nephrology for patients with CKD) is available from November 2023. A slight majority of providers (52%) are facilities with less than 5,000 patients declared (Supplementary Chart S1). The analysis of contract implementation in 2023 indicates that over 2 million services were settled for a total amount exceeding 220 million PLN (Polish Zloty; 51, 3 million EUR) (51). The implementation of the new care model by 2,947 facilities by the end of 2025, covering 49% of PHC facilities in Poland, highlights substantial progress. The increased access to these services for 61% of the population reflects the model's expanding reach and its potential for greater healthcare inclusivity.

The implementation of CC resulted in an increase in the number of patients receiving services with ICD-10 diagnoses for disease groups included in CC, with most pathways having only half of new diagnoses recorded in facilities included in CC (Supplementary Table S2). It should be noted, however, that there was a decline in 2020, likely due to the COVID-19 pandemic (51).

A particularly notable increase in the number of diagnoses of CKD in the 1st year of CC implementation. In the 1st year, the number of patients visiting facilities providing CC with an ICD-10 code for CKD (N18) increased from 69,710 to 94,056, representing almost a 35% increase (Supplementary Chart S2) (51). These observations suggest an early positive impact of the model's adoption on chronic disease recognition.

The expansion of CC increases demands on PC providers, particularly family physicians, who may face burnout due to higher patient volumes and added administrative tasks. Effective strategies, such as better administrative support and recruitment, are necessary for long-term success.

### 4.3 Strengths and limitations of the review

This work is based on a literature review with clearly specified criteria outlined in the introduction. At the same time, this review aimed primarily to indicate the scope of PHC services in line with the CC model in Poland and is not a systematic review of all PHC models in European systems. Not all countries have sufficient English-language literature to generalize conclusions about the organization and actual implementation of PC models. Although the focus of the article was on the relevance and comprehensiveness of the studies rather than their individual methodological quality, this decision may limit the ability to draw firm conclusions about the reliability and validity of the data presented in the review. Future reviews could benefit from a more structured quality assessment to better evaluate the strength of the evidence.

A narrative review can descriptively identify the organizational elements of healthcare systems at the primary level in various European countries. However, compilations of results are difficult due to differences in both the organization of the systems and the reporting of activities. This also poses a significant challenge for organizations such as the WHO, the European Commission, and the Organization for Economic Co-operation and Development. It is easier to compare data related to morbidity and health risks, while comparing the impact of healthcare organizations on medical outcomes is more difficult. Analysis between countries is also difficult due to the diversity of data.

The CC model was effectively introduced at the beginning of 2023, so facilities have been operating based on it relatively briefly. However, the utilization of resource allocation and the voluntary participation of almost 50% of PC facilities in this model in Poland allows for initial generalizations and summaries of the implementation itself. Furthermore, the use of publicly available data from sources such as the National Health Fund and the Ministry of Health may be limited in scope and completeness, as it excludes unpublished or internal data that could provide a more comprehensive picture of the model's effectiveness. To determine the limitations of the CC model itself, further analyses of the system introduced in Poland and comparisons to foreign systems are necessary, both in a time extension and in terms of population health needs. This should be the subject of further research. These studies should also examine the impact of specific actions or omissions by the system organizer (payer) on the implementation of health policy within PHC, particularly in terms of piloting, training before and during implementation, and support for the facilities themselves.

While the data indicates an increase in service use and diagnostic rates, it is important to recognize that these trends may reflect both over diagnosis or the effect of incentivization of providers rather than purely improved health outcomes. Additionally, reporting biases, such as an overrepresentation of certain chronic conditions, may skew the perceived success of the model. A more thorough evaluation, including long-term follow-ups and patient outcomes, will be required to assess the model's impact.

However, given these early results, it is reasonable to conclude that such models can enhance chronic disease management in PHC settings. It is crucial to emphasize that these improvements should be cautiously interpreted, considering the potential risks of reporting bias. A robust evaluation framework is essential to fully understand the model's long-term effects.

## 5 Conclusions

This narrative review has systematized the role of the PHC system in addressing now-a-day demographic-epidemiological challenges and preliminary data indicates that:

- There is potential to utilize existing resources within the PHC in healthcare systems. The use of pilot programs, the creation of an incentive system, and voluntary-based implementation could serve as a model supporting such utilization.
- A well-conducted pilot and implementation allow for the initiation of a new care model based on voluntary participation of providers. This approach allows the parties (including PHC teams) involved in the implementation to maintain autonomy and enables them to retain influence over the final outcome of the implementation.
- A care model based on fee-for-service (FFS) financing can be introduced even in systems that previously lacked such a service model. Such implementation, preceded by a pilot and educational activities, should involve not only the engaged medical staff but also the support personnel in PHC.

The systemic change in Poland is a good example that it is possible to expand the systemic powers of a PC physician based on their competencies, build PC teams, and designate new roles in the PHC system, including by introducing new personnel to PC.

A CC model that involves not only the PC physician but also other PC team members and other specialists allowed to utilize the resources for comprehensive patient-centered care. The Polish model emphasizes enhanced diagnostic capabilities, interdisciplinary collaboration, and patient education within PHC teams, although these elements should be assessed carefully and with taking into account demographic changes, morbidity causes and financial factors in the healthcare system. Key innovations in CC in Poland include Individual Health Care Plans (IHCPs), expanded diagnostic tools, and a new coordinator role to streamline patient pathways—each of these elements potentially supports the patient in accessing services—although this also requires further analysis.

The sustainability of the CC model remains uncertain, as the fee-for-service financing may become financially unsustainable without long-term support. Future assessments should explore how to maintain financial viability and avoid widening disparities between healthcare facilities.

While CC aims to improve access, it is crucial to examine whether all patient populations, especially vulnerable groups, benefit equally. More research is needed to assess if the model addresses disparities or if some populations remain underserved.

Early data indicate increased provider participation and improved chronic disease diagnostics, especially for conditions like CKD. However, challenges remain, including integration with existing services. The study underscores CC's potential to enhance healthcare quality through strengthened PHC, while emphasizing the need for further evaluation of its long-term impact and adaptability across systems.
