# Supplementary material for: Coordinated Care: the new model in primary health care in Poland—Implementation and early trends
Source: Front Public Health. 2026 Apr 15;14:1737980. doi: 10.3389/fpubh.2026.1737980 (PMC13124697; doi:10.3389/fpubh.2026.1737980)
Supplement: Supplementary file 1 [file Table_1.docx]

**Supplementary Table S1. Number of Doctor-Doctor and Doctor-Patient Consultations Provided as Part of Coordinated Care (Percentage of the Population with Comprehensive Consultation in Brackets)**

|  | Doctor-Doctor Consultations | Doctor-Patient Consultations |
| --- | --- | --- |
| Cardiology consultation | 3 649 (0,61%) | 61 820 (10,37%) |
| Endocrinology consultation | 1 347 (0,23%) | 20 099 (3,37%) |
| Diabetology consultation | 991 (0,17%) | 19 209 (3,22%) |
| Pulmonology consultation | 263 (0,04%) | 6 965 (1,17%) |
| Allergology consultation | 22 (0,00%) | 507 (0,09%) |
| Nephrology consultation | 7 (0,00%) | 37 (0,01%) |
