# Supplementary material for: Coordinated Care: the new model in primary health care in Poland—Implementation and early trends
Source: Front Public Health. 2026 Apr 15;14:1737980. doi: 10.3389/fpubh.2026.1737980 (PMC13124697; doi:10.3389/fpubh.2026.1737980)

**Supplementary Chart S1. Healthcare Providers with a Contract Period from January to October 2023 for the Budget Entrusted to Coordinated Care and the Number of Registered Patients**


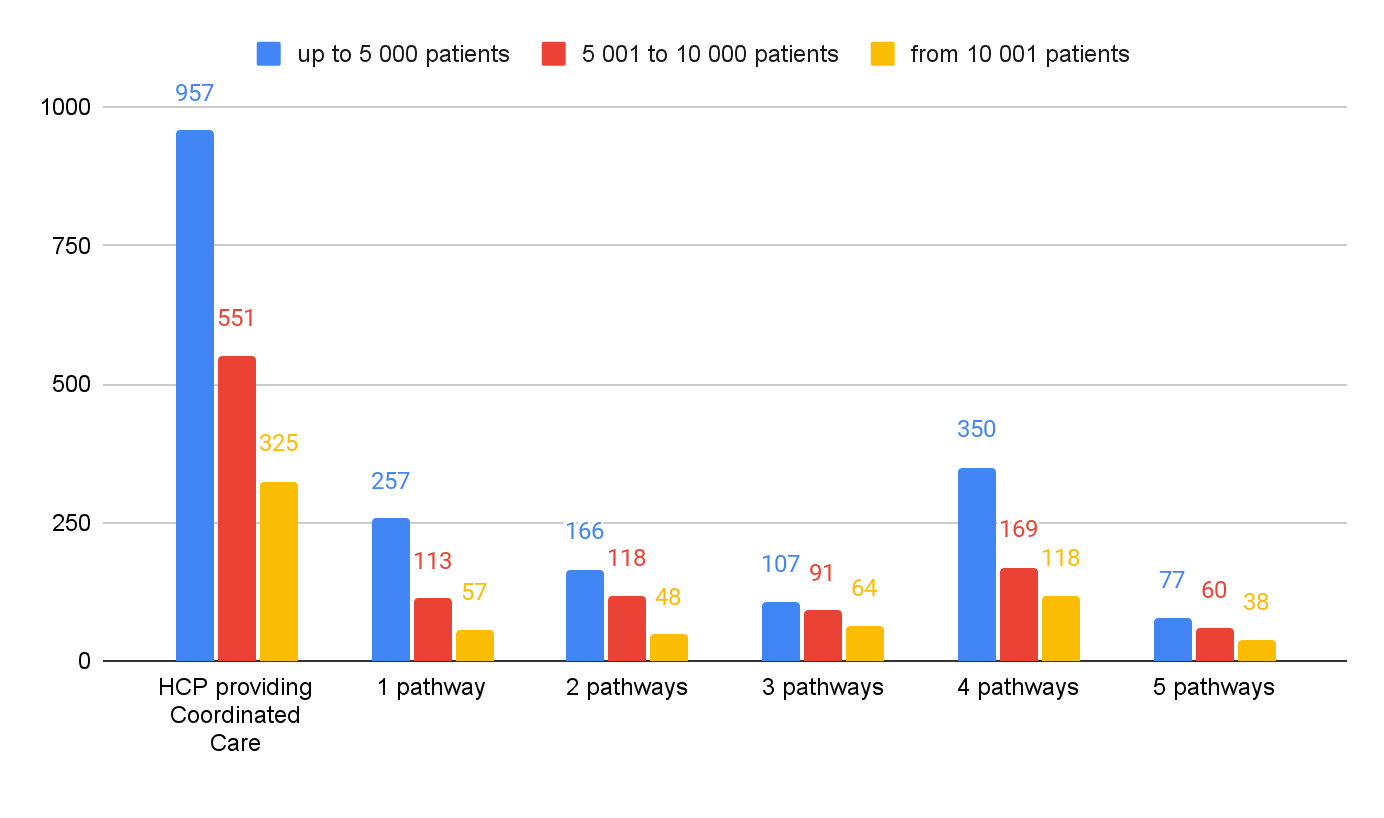

Supplement: Supplementary file 2 [file Data_Sheet_1.docx]
