# Supplementary material for: Coordinated Care: the new model in primary health care in Poland—Implementation and early trends
Source: Front Public Health. 2026 Apr 15;14:1737980. doi: 10.3389/fpubh.2026.1737980 (PMC13124697; doi:10.3389/fpubh.2026.1737980)

**Supplementary Chart S2. Number of Patients at Primary Care Providers Included in Coordinated Care Who Had a Primary Care Physician Service with a Primary or Co-occurring Diagnosis of CKD (N18 According to ICD-10) in 2019-2023**


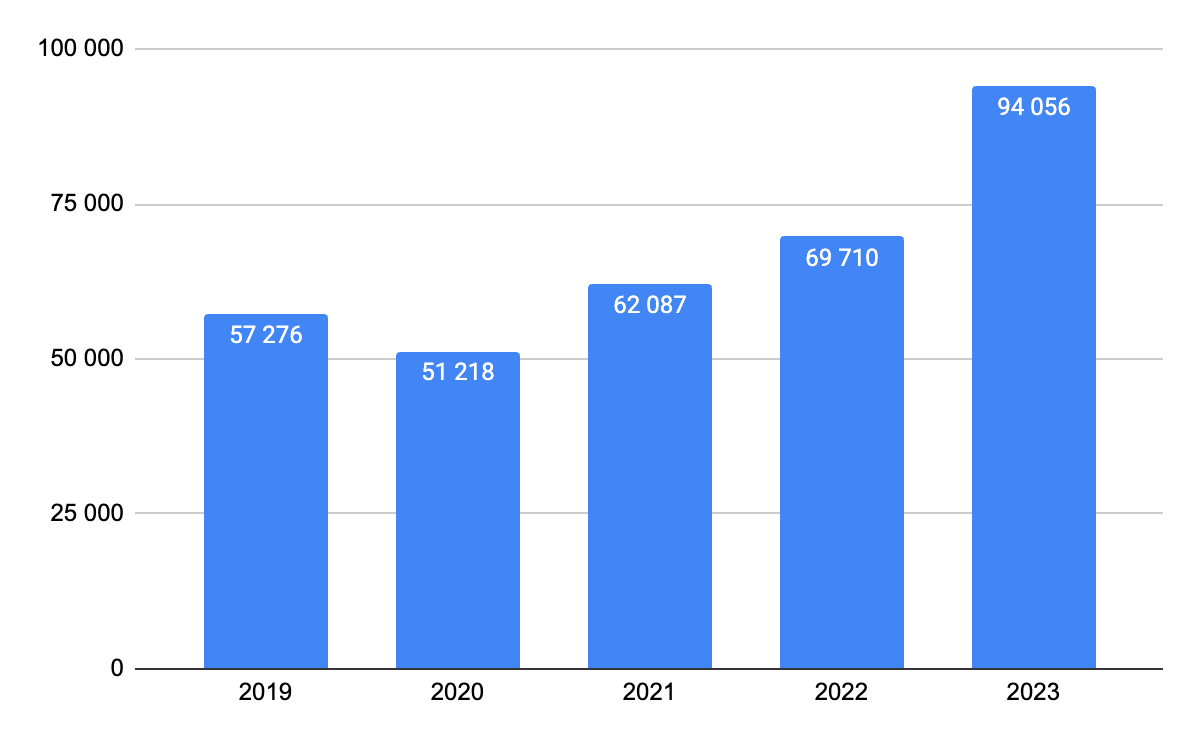

Supplement: Supplementary file 3 [file Data_Sheet_2.docx]
